# tttrlib: modular software for integrating fluorescence spectroscopy, imaging, and molecular modeling

**DOI:** 10.1093/bioinformatics/btaf025

**Published:** 2025-01-21

**Authors:** Thomas-Otavio Peulen, Katherina Hemmen, Annemarie Greife, Benjamin M Webb, Suren Felekyan, Andrej Sali, Claus A M Seidel, Hugo Sanabria, Katrin G Heinze

**Affiliations:** Department of Bioengineering and Therapeutic Sciences, Department of Pharmaceutical Chemistry, and Quantitative Biosciences Institute, University of California, San Francisco, CA, 94143, United States; Rudolf Virchow Center for Integrative and Translational Bioimaging, Julius-Maximilians-University Würzburg (JMU), Würzburg, 97080, Germany; Rudolf Virchow Center for Integrative and Translational Bioimaging, Julius-Maximilians-University Würzburg (JMU), Würzburg, 97080, Germany; Chair of Molecular Physical Chemistry, Heinrich-Heine University, Düsseldorf, 40225, Germany; Department of Bioengineering and Therapeutic Sciences, Department of Pharmaceutical Chemistry, and Quantitative Biosciences Institute, University of California, San Francisco, CA, 94143, United States; Chair of Molecular Physical Chemistry, Heinrich-Heine University, Düsseldorf, 40225, Germany; Department of Bioengineering and Therapeutic Sciences, Department of Pharmaceutical Chemistry, and Quantitative Biosciences Institute, University of California, San Francisco, CA, 94143, United States; Chair of Molecular Physical Chemistry, Heinrich-Heine University, Düsseldorf, 40225, Germany; Department of Physics & Astronomy, Clemson University, Clemson, SC, 29634, United States; Rudolf Virchow Center for Integrative and Translational Bioimaging, Julius-Maximilians-University Würzburg (JMU), Würzburg, 97080, Germany

## Abstract

**Summary:**

We introduce software for reading, writing and processing fluorescence single-molecule and image spectroscopy data and developing analysis pipelines to unify various spectroscopic analysis tools. Our software can be used for processing multiple experiment types, e.g. for time-resolved single-molecule spectroscopy, laser scanning microscopy, fluorescence correlation spectroscopy and image correlation spectroscopy. The software is file format agnostic and processes multiple time-resolved data formats and outputs. Our software eliminates the need for data conversion and mitigates data archiving issues.

**Availability and implementation:**

*tttrlib* is available via pip (https://pypi.org/project/tttrlib/) and bioconda while the open-source code is available via GitHub (https://github.com/fluorescence-tools/tttrlib). Presented examples and additional documentation demonstrating how to implement *in vitro* and live-cell image spectroscopy analysis are available at https://docs.peulen.xyz/tttrlib and https://zenodo.org/records/14002224.

## 1 Introduction

Making raw intensity images accessible is an endeavor addressed by the tagged-image file format (TIFF), open-microscopy environment (OME) TIFF (Goldberg *et al.* 2005) and, more recently, by OME-Zarr (Moore *et al.* 2023). There are two challenges: (i) metadata necessary to interpret the original data must be accessible and preserved, and (ii) original data must be readable. The development of our software for time-resolved single-molecule and imaging data was motivated by integrative modeling requirements (Hancock *et al.* 2022) and the need for standard data interfaces and automated analysis pipelines. The lack of vendor-independent time-resolved single-photon data formats was recognized by the FRET community (www.fret.community), a scientific community founded to enhance dissemination and community-driven development of analysis tools (Lerner *et al.* 2021), as a challenge for fluorescence-based integrative models, as the archiving system for integrative models (Burley *et al.* 2017) recommends to archive models along with relevant experimental data, metadata, and experimental/computational protocols (Berman *et al.* 2019, Hanke *et al.* 2024). In single-molecule Förster resonance energy transfer experiments (smFRET) the lack of data formats was mitigated by a Hierarchical Data Format (HDF5) based format (Ingargiola *et al.* 2016b). In the past, our software led to publications with a focus on spectroscopy (Hemmen *et al.* 2021, Balakrishnan *et al.* 2022) and image spectroscopy (Liu *et al.* 2023). Our software provides a unified interface to diverse proprietary data formats. It solved archiving and storing issues at our imaging facility and makes data accessible to customers.

**Figure 1.**
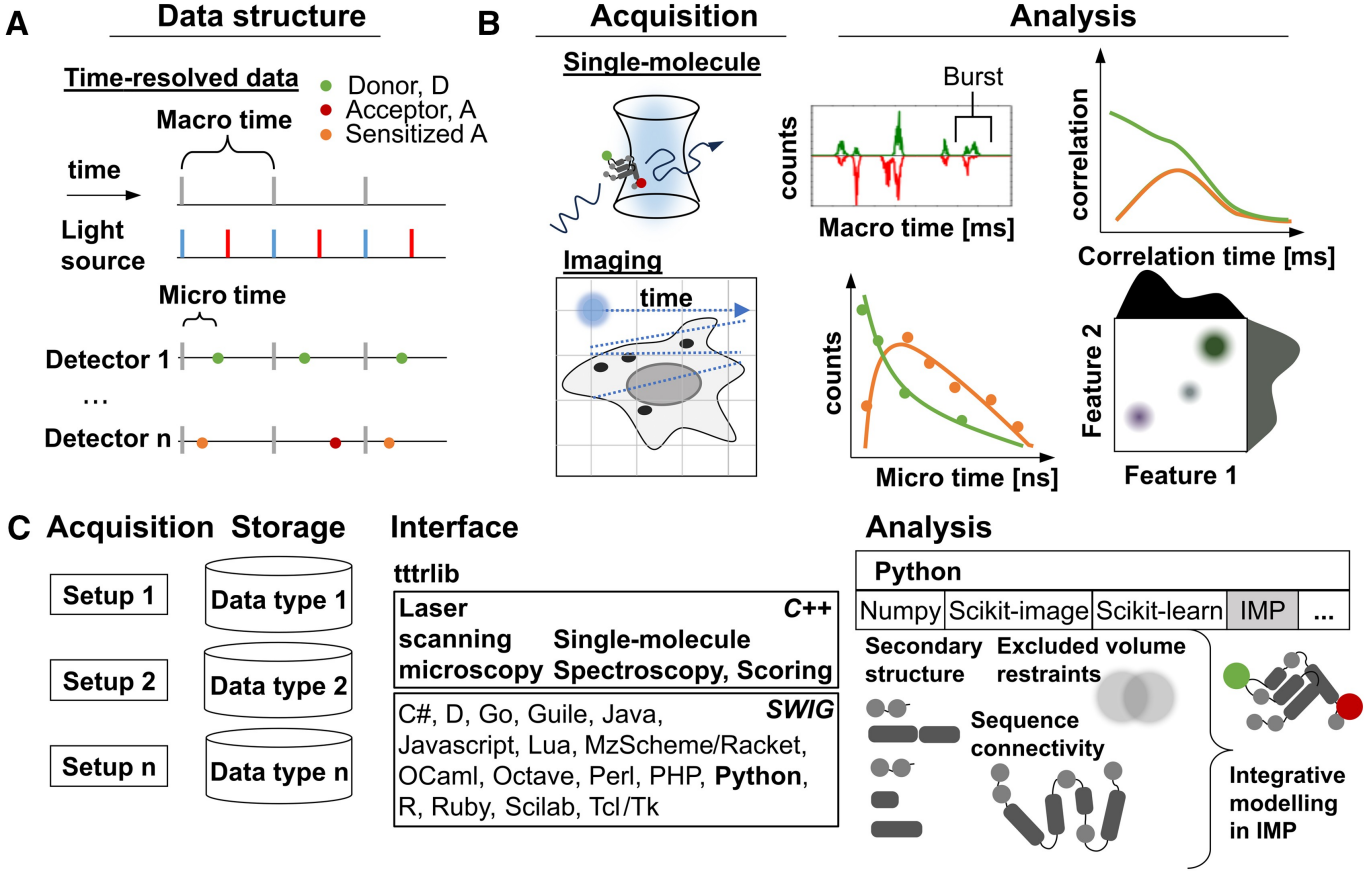
Time-resolved single-molecule fluorescence spectroscopy (SMS) and fluorescence image spectroscopy (FIS) share data registration and processing routines. (A) In time-resolved fluorescence, excitation and photon detection events are encoded using a macro and a micro time counter. In pulsed experiments the coarse macro time counter is synchronized to the light source. The micro time measures the time delay relative to a macro time event (laser pulse). Events are characterized by an identifier, usually referring to the detection channel or TCSPC board input number. (B) Confocal SMS registers photons of freely diffusing molecules in a stationary excitation and detection volume. Confocal FIS generates an image by sweeping an excitation source over a sample and registering the emitted photons over time in a photon stream. SMS and FIS process macro and micro times. In SMS, photons are grouped into single-molecule events. FIS groups photons into pixels. Macro/micro times of photons in a group are analysed to determine spectroscopic features (e.g. fluorescence lifetimes or anisotropies). (C) Our C++ library *tttrlib* is wrapped for scripting languages via the Simplified Wrapper and Interface Generator (SWIG) and abstracts data to provide an interface to data, methods, and algorithms for laser scanning microscopy and single-molecule spectroscopy. This enables fluorescence data analysis by diverse sources using other data processing and analysis libraries such as scikit-image and scikit-learn and integrative modeling by the Integrative Modeling Platform (IMP). The acquired information/data is interpreted by modeling that can consider additional information, e.g. secondary structure, excluded volume or sequence connectivity information to produce a structural integrative model.

Compared to intensity-based experiments, time-resolved spectroscopy and spectroscopic imaging data are more complex since spectroscopic and image information are jointly encoded (Fig. 1A). Such information can be used in FRET and photo-induced electron transfer (PET) experiments to map distances between fluorophores in the range from 1 to 10 nm at Ångstrom resolution (Stryer 1978, Clegg *et al.* 1993, Bates *et al.* 2005, Sahoo *et al.* 2007) and inform on dynamics of molecular processes in solution (Kalinin *et al.* 2012, Barth *et al.* 2022, Opanasyuk *et al.* 2022) and in living cells. While fluorescence correlation spectroscopy (FCS) (Elson and Magde 1974) combined with FRET or PET can map distances as a function of time over 10 time decades with sub-nanosecond resolution (Böhmer *et al.* 2002, Kapusta *et al.* 2007, Felekyan *et al.* 2012), fluorescence experiments are sufficiently sensitive (Moerner and Kador 1989, Orrit and Bernard 1990, Shera *et al.* 1990) to study single molecules for disentangling complex biomolecular systems (Ha *et al.* 1996, Weiss 1999). Fluorescence spectroscopy combined with microscopy informs on large biomolecular assembly structures either *in vitro* (Peulen *et al.* 2023, Kolimi *et al.* 2024) or in living cells (Jares-Erijman and Jovin 2003, Sakon and Weninger 2010, Fessl *et al.* 2012, Stahl *et al.* 2013, König *et al.* 2015, Kravets *et al.* 2016) at any size, ranging from polyproline oligomers (Stryer 1978, Schuler *et al.* 2005, Best *et al.* 2007, Hoefling *et al.* 2011) to ribosomes (Hickerson *et al.* 2005) and can map interactions in living cells at high-throughput (Ries *et al.* 2010, Wachsmuth *et al.* 2015).

Single molecule spectroscopy (SMS) and fluorescence imaging spectroscopy (FIS) rely on the time-resolved registration of photons. FIS and SMS data are encoded as photon streams (Fig. 1A). In SMS, fluorescence bursts are selected by intensity thresholding the background. In FIS, the data stream is sorted into pixels of an image. In both cases, photons grouped into bursts or pixels need to be analysed, e.g. by counting, distribution or correlation analysis. Frequently bursts and pixels are grouped by spatial (e.g. shapes in images), temporal (e.g. kymographs in time-series analysis), or spectroscopic features (e.g. fluorescence lifetimes) into sub-ensembles and regions, respectively. SMS and FIS share similar challenges, as the number of photons in a pixel or burst is low (in the order of 10’s to hundreds of photons). Thus, identical analysis routines are used for apparently different tasks (Fig. 1B). Nevertheless, even though data storage and processing routines of SMS and time-resolved FIS are largely identical, to the best of our knowledge, no established unified framework exists for FIS and SMS data.

Therefore, we introduce a software that preserves metadata and gives access to raw data of proprietary data formats (e.g. PicoQuant, Becker&Hickl, Zeiss, Leica) via a unified interface. Our software reads and processes spectroscopic and image data for downstream processing and for integrating experimental data into analysis frameworks such as the Integrative Modeling Platform (Russel *et al.* 2012) (IMP) (Fig. 1C). Examples available at https://docs.peulen.xyz/tttrlib illustrate how to work with our library. Here, we present an SMS workflow that processes human Guanylate binding protein 1 (hGBP1) single-molecule FRET data by burst-integrated fluorescence lifetime analysis (BIFL), fluorescence correlation spectroscopy (FCS) and photon distribution analysis (PDA) (Supplementary Fig. S1). Finally, we preprocess murine guanylate binding protein (mGBP) data in a workflow that combines classic image analysis with spectroscopy (Supplementary Fig. S2).

## 2 Implementation

*tttrlib* is a file format-independent interface to FIS and SMS data that mimics the expected behavior of common numeric libraries such as NumPy (https://www.numpy.org). The functionalities were implemented following design guidelines for operations on sequence and list data in scripting languages such as slicing of data objects. tttrlib objects are initialized via keyword arguments. This allows conveniently creating and archiving settings used in analysis in nonrelational object databases or dictionary files (e.g. JSON, YAML, or mmCIF).

The time-tagged time-resolved (TTTR) data is accessed by the TTTR class. TTTR objects can be sliced and merged to facilitate or distribute the processing of larger datasets. Slicing and merging TTTR objects allows users to build filters to select events by micro- or macro-time counter values, e.g. to discriminate depletion pulses in STED microscopy or molecular aggregates in single-molecule spectroscopy. Metadata of TTTR objects is accessed via a Header class and dictionaries. Modifying the metadata and saving TTTR objects to other file types enables cross-compatibility of manufacture-specific analysis software. Selection algorithms allow defining ranges based on average intensities in a time window, and detector numbers can be used to partition data. For the common operations (intensity thresholds, selection of detection channels, etc.) selection algorithms are predefined to select subsets of the data.

*Requirements.*  *tttrlib* is available and tested on all major operating systems (Linux, macOS, and Windows). It can be installed in conda and scripted via Python.

*Supported microscopes and data formats. tttrlib* can be used for confocal laser scanning microscopy (CLSM) TTTR data and implements reading routines for the most common CLSM microscopes (such as the Leica SP5/SP8, Zeiss LSM980). So far, *tttrlib* supports reading of the proprietary data format of Picoquant (PTU/HT3), Becker&Hickl (SPC-130, SPC-630), Zeiss (confocor), the single-molecule SM file format, and the open source PhotonHDF5 format. The most common parameters necessary for interpreting CLSM TTTR data can be user-specified. Thus, *tttrlib* can be used to process arbitrary TTTR and time-resolved CLSM data.

## 3 Results

### 3.1 Spectroscopy

Our software offers high- and low-level processing and analysis methods that can be combined into pipelines for developing new ensemble fluorescence spectroscopy, SMS, and FIS methodologies. A features subset is demonstrated in a single-molecule analysis of human guanylate binding protein (hGBP1) smFRET data (Supplementary Fig. S1). In the confocal experiments, fluorescence of dilute freely diffusing labeled hGBP1 was registered (Peulen *et al.* 2023). The analysis pipeline (i) reads smFRET data, (ii) selects single-molecule events (Fries *et al.* 1998, Schaffer *et al.* 1999, Maus *et al.* 2001), (iii) performs a burst analysis (computes intensity and lifetime-based FRET indicators), (iv) correlates photon traces, (v) generates single-molecule counting histograms, analyzed by (vi) photon distribution analysis (PDA) (Antonik *et al.* 2006) and burst variance analysis (BVA) (Torella *et al.* 2011), and (vii) selects molecular sub-ensembles. Details are described in the Supplementary Methods and Supplementary Results.

### 3.2 Image fluorescence spectroscopy

Our image spectroscopy (FIS) pipeline (Supplementary Fig. S2) processes time series acquired on MEF cells transfected with murine guanylate binding proteins 3 (mGBP3) and mGBP7 N-terminally tagged with mCherry and eGFP, respectively. GBPs localize in the cytoplasm and accumulate in vesicle-like structures (VLS) (Kravets *et al.* 2016). The pipeline (i) groups photons into pixels of intensity images, (ii) performs a FLIM analysis for multiple detection channels and excitation sources, (iii) segments images into pixel classes, (iv) uses the model-free phasor approach to highlight sample heterogeneity (Digman *et al.* 2008), and (v) extracts fluorescence decays of pixel-classes for sub-ensemble analysis. The details are described in the Supplementary Methods and Supplementary Results.

## 4 Discussion

We introduced open-source software for processing various time-resolved data via a unified interface. Our software *tttrlib* integrates into various scripting languages for simple usage for custom data processing pipelines and automated FIS or SMS data processing. Our abstraction layer to vendor-specific original data and metadata addresses the challenge of preserving and processing data independent of its origin (Fig. 1C). The customizable vendor-agnostic reading routines process data of multiple experimental setups of varying types without the need for data conversion. By operating on original data, we assert that metadata essential for handling microscopy data is preserved. Our software is programmed in C/C++ and comes with a set of algorithms and tools that operate on the ingested data. It was tested for single-molecule (Supplementary Fig. S1) and image data (Supplementary Fig. S2) registered by multiple excitation and detection modalities, including pulsed interleaved excitation (PIE) (Müller *et al.* 2005, Hendrix and Lamb 2013) and multiparameter fluorescence detection (MFD) (Sisamakis *et al.* 2010) for studying protein structures and dynamics *in vitro* (Hellenkamp *et al.* 2018, Lerner *et al.* 2021) and in living cells (Weidtkamp-Peters *et al.* 2009, Stahl *et al.* 2013). *tttrlib* provides the most commonly used data reduction and analysis algorithms of fluorescence spectroscopy such as PDA (Antonik *et al.* 2006) and correlation algorithms for FCS (Wahl *et al.* 2003) that can be used for advanced FCS techniques such as full-, gated-, or filtered-FCS and methods for time-resolved image spectroscopy and image correlation spectroscopy (ICS) (Petersen *et al.* 1993). Here, we presented the most common single-molecule spectroscopy and imaging modalities applications. Applications for less commonly used methods (e.g. ICS) are presented in the online repository.

We wrapped our software using the Simplified Wrapper and Interface Generator (SWIG) for simple integration into scripting and other programming languages. Currently we focus on the most widely used programming language Python. Integrating our software into Python enables the use of time-resolved data in data-science software packages such as scikit-image (van der Walt *et al.* 2014), scikit-learn (Pedregosa *et al.* 2011) and the Integrative Modeling Platform (IMP) (Russel *et al.* 2012). For easy construction of custom analysis pipelines, we implemented our software in a fully modular fashion (Fig. 1C). This is illustrated by Python implementations of example smFRET and FIS analysis pipelines and more extensive documentation, benchmarks and examples in the online documentation. These code examples can serve as templates to develop custom analysis pipelines and data processing workflows.

Various FIS and SMS software (lifetime fits and phasor analysis) exist. FLIMJ, based on the FLIMlib library, focuses on ease of use by providing graphical user interfaces (GUI) for basic FIS analysis in ImageJ for Linux, macOS and Windows (Gao *et al.* 2020). FLIMfit offers many model functions for FIS but lacks, as stand-alone software, a close integration with image analysis (Warren *et al.* 2013). General SMS software such as PAM (Schrimpf *et al.* 2018) and software maintained by single-molecule labs usually offer ease-of-use through GUIs and a large toolbox for FIS and SMS (Weidtkamp-Peters *et al.* 2009, Sisamakis *et al.* 2010). This software is often based on closed-source programming languages, lacks scripting capabilities or interfaces to other toolboxes (e.g. for machine learning), and is tightly integrated with GUIs (Schrimpf *et al.* 2018). A collection of SMS software is compiled and maintained by the FRET community (see: https://fret.community/software/). More recent open-source SMS software such as PhotonHDF and FRETBursts mitigate the absence of open standards in SMS and give scripting capabilities and interfaces to other toolboxes; however, they require a conversion of the original data to an open format (Ingargiola *et al.* 2016a) and lack imaging capabilities. Our modular library was developed for programming analysis workflows while most existing FIS and SMS software was designed for established data processing workflows and user-specific cases. By focusing on developers and data scientists, *tttrlib* can be closely integrated with other software such as NumPy/SciPy, scikit-image, or IMP.

## Supplementary Material

btaf025_Supplementary_Data

## Data Availability

Presented examples and additional documentation demonstrating how to implement in vitro and live-cell image spectroscopy analysis are available at https://docs.peulen.xyz/tttrlib and https://zenodo.org/records/14002224.
