# Supplementary material for: tttrlib: modular software for integrating fluorescence spectroscopy, imaging, and molecular modeling
Source: Bioinformatics. 2025 Jan 21;41(2):btaf025. doi: 10.1093/bioinformatics/btaf025 (PMC11796090; doi:10.1093/bioinformatics/btaf025)
Supplement: btaf025_Supplementary_Data [file btaf025_supplementary_data.zip › btaf025_Supplementary_Data.zip!/8062f_250103_tttrlib_supplement.pdf]

Supplementary Information

tttrlib: modular software for integrating fluorescence spectroscopy, imaging, and molecular modeling

Thomas-Otavio Peulen[1,2,3,4,*#], Katherina Hemmen[4,*], Annemarie Greife[5], Benjamin M. Webb[1,2,3], Suren Felekyan[5], Andrej Sali[1,2,3], Claus A. M. Seidel[5], Hugo Sanabria[6], Katrin G. Heinze[4#]

[1]Department of Bioengineering and Therapeutic Sciences, University of California, San Francisco, San Francisco, California, United States; [2]Department of Pharmaceutical Chemistry, University of California, San Francisco, San Francisco, California, United States; [3]Quantitative Biosciences Institute (QBI), University of California, San Francisco, San Francisco, California, United States; [4]Rudolf Virchow Center for Integrative and Translational Bioimaging, University of Würzburg, Würzburg, Germany; [5]Chair of Molecular Physical Chemistry, Heinrich-Heine University, Düsseldorf, Germany; [6]Department of Physics & Astronomy, Clemson University, Clemson, SC 29634, USA;

* First author

# Corresponding author

Keywords: Fluorescence spectroscopy, fluorescence microscopy, integrative modeling, structural biology, single-molecule FRET

Supplementary Materials and Methods

smFRET data set

Sample preparation and data acquisition

We process data of the ligand free non-farnesylated human guanylate binding protein 1 (hGBP1) (*1*). Larger single-molecule FRET datasets on hGBP1 are openly available (https://zenodo.org/records/6534557). Briefly, cysteines were introduced at specific positions in a cysteine-free hGBP1 variant. Here, we process data of the cysteine mutant Q344C-Q525C where we attached Alexa488 and Alexa647 via maleimide labeling chemistry (*1*).

Burst-wise photon selection and analysis

We select the photons emitted from the freely diffusing labeled protein as fluorescence intensity peaks ("bursts") by thresholding against a minimal number of photons in a defined time window. Here we applied a minimal threshold of 60 photons per burst. The macro- and microtime of the selected photons are saved and grouped into bursts. Based on the selected bursts, the spectroscopic properties like countrate in green or red channels, fluorescence-weighted mean fluorescence lifetime or scatter-corrected anisotropy are determined. Additionally, parameters like the average FRET efficiency can be derived from the directly determined spectroscopic parameter.

Count-rate selective fluorescence correlation spectroscopy

In single-molecule experiments a large amount of photons stems from uncorrelated scatter contribution, *e.g.*, the measurement buffer. This reduces the correlation amplitude. We correlate the whole photon stream and to secondly correlate only high count rate sections of the continuous data stream. Here, we specified that more than 60 photons have to be present in a 10 ms time window for the photons collected during this time window to be included in the correlation routine. The selection procedure is applied to the whole data set, i.e. photons are summed over all eight measurement channels, however, the correlation routine is selectively applied to either correlate the green (pG/sG) or the red channels only (pR/sR) or to correlate the green channels with the red channels (pG,sG/pR,sR).

Photon Distribution Analysis (PDA)

For photon distribution analysis, the intensity traces were sliced into time windows (TW) of 1 ms, for TWs with at least 20 photons, the green-to-red intensity ratio Sg/Sr was calculated and Sg/Sr experimental frequency histograms generated. Here, we optimize parameters of model frequency histograms of a two state or three state model. Our models additionally consider a donor-only population. In our example, we fit Sg/Sr experimental histograms by optimizing the model parameters with routines provided by scipy. *tttrlib* provides programmable PDA model outputs. First, the 1 dimensional representation of experimental two dimensional counting data (red / green, parallel / perpendicular) can be freely specified. Thus also anisotropy data can be optimized or sampled. Second, the forward model, i.e, the model that is used to compute experimental observables (such as Sg/Sr), is programmable. Thus, arbitrary two dimensional counting data can be described by complex model functions.

Burst Variance Analysis (BVA)

We demonstrate an implementation of burst variance analysis (BVA) to estimate conformational dynamics in single-molecule Förster resonance energy transfer (smFRET) experiments. In BVA, the variance of the proximity ratio is calculated for each single-molecule burst, and dynamics are detected if this variance surpasses the shot noise limit (*2*). This standard deviation is then plotted against the average proximity ratio, with the shot noise limit—determined by photon count—providing a lower boundary. Events exceeding this boundary are classified as dynamic, indicating potential conformational changes.

Please note, that detection of dynamics is inherently limited by the count rate (*e.g.*, 100 kHz per molecule), which can prevent observation of fast conformational dynamics. To overcome this, we additionally estimate variance using fluorescence lifetimes (which are independent of count rate) and FRET efficiency (not shown). This approach avoids the need for sub-sampling within each burst and, therefore, remains unaffected by count rate constraints, allowing more accurate detection of rapid dynamics (*3*).

Image spectroscopy data set

Sample preparation

Measurements were performed in MEF mGBP7 deficient cells stably transduced with eGFP-mGBP7 and mCh-mGBP3. Generation, culture conditions and characterization of the cell line is described in (*4*, *5*). MEF cells were seeded in fully supplemented DMEM medium and grown until 70-80% confluence in Nunc™ LabTek™ II 8-well chambers (ThermoFisher). For live cell pulsed-interleaved excitation (PIE) MFIS-FRET measurements, the medium was changed to pre-warmed FluoroBrite™ DMEM (Gibco). Cells were kept at 37°C during the measurements.

PIE measurement

PIE experiments were performed on a confocal laser-scanning microscope (FV1000 Olympus, Hamburg, Germany) equipped with a single photon counting electronics with picosecond time-resolution (HydraHarp 400, PicoQuant, Berlin, Germany). eGFP was excited at 488 nm with a polarized, pulsed 20 MHz diode laser (LDH-D-C-485, Pico-Quant, Berlin, Germany) using a power of 28 nW at the objective. mCherry was excited at 565 nm with a white light laser with a 20 MHz repetition rate (NKT) using a power of 175 nW at the objective. The emitted light was collected through the same objective and separated into perpendicular and parallel polarization. A narrow range of eGFPs emission spectrum (bandpass filter: HC520/35, AHF, Tübingen, Germany) was then detected by single photon avalanche detectors (PDM50-CTC, Micro Photon Devices, Bolzano, Italy). mCherry fluorescence was detected by hybrid detectors (HPMC-100-40, Becker&Hickl, Berlin, Germany, with custom designed cooling). The mCherry detection wavelength range was set by bandpass filters (HC 609/54, AHF). To measure a single cell, we chose a 256x256 pixel ROI and collected 400 frames per image with 4 μs dwell time.

Donor mean fluorescence lifetime and Phasor plot

We calculate the donor mean fluorescence lifetime and perform phasor analysis (*6*) of the stacked image frames. In both cases, we only consider the green channels in the prompt time window of the PIE measurement and correct for the IRF. The donor mean fluorescence lifetime

is calculated for all pixels with at least 20 photons in sum, while for the phasor plot only pixels with at least 30 photons in sum were used.

Image segmentation

We segmented the intensity images frame-wise into three pixel classes, nucleus, cytoplasm and vesicle-like structures (VLS), using scikit-image (*7*). For segmentation of VLS and cytoplasm, the intensity sum of the green and red channels was used. For segmentation of the cytoplasm, a median filter with three pixel radius was applied to the image frames followed by thresholding using Otsu's method (*8*). Next, small holes in the cytoplasm were filled and fragments of neighboring cells removed by selecting for a minimal area. VLS were identified from surrounding cytoplasm by Gaussian smoothing of the image frames with a single pixel radius followed by Otsu thresholding. Only identified pixel regions larger than five pixels were considered as VLS. Finally, the region of the nucleus was defined. Here, the image frames of the green channel were first smoothed using a Gaussian filter with three pixel radius, followed by thresholding using Li's method (*9*, *10*). The thresholded image was dilated, holes inside the nucleus filled and small areas outside removed. Next all left-overs outside the cytoplasm, i.e. from neighboring cells, were removed by multiplying with the generated cytoplasm mask, followed by another round of dilation to make the nucleus "smoother". Finally, we generated an average nucleus by summing over all 400 frames and thresholded this projection using the method of Otsu. Next, the three masks were combined to assign each pixel in each frame mutually exclusive to a single class, i.e. all pixels inside the cytoplasm mask which belong to the VLS or nucleus were removed from the cytoplasm mask.

Pixel-wise analysis

The pixel masks for nucleus, cytosol and VLS were in the next step used to select the photons present in the respective regions of interest. For the photon arrival histogram we summed the microtimes of all photons present in the ROI up while additionally selecting for the detection channels (green / red) and the time window (prompt / delay). Additionally, we evaluated per pixel in each of those ROIs the fluorescence spectroscopy parameter such as intensity ratios, effective stoichiometry, S_{PIE}, or the proximity ratio, PR. To achieve this, we simply multiplied

our binary masked (consisting of 0's and 1's) with the intensity, fluorescence lifetime or phasor images exported above. The resulting parameters were written into a table for easy construction of one- or two-dimensional histograms using any data analysis software. Of note, please be aware that no correction factors such as direct acceptor excitation or donor crosstalk in the acceptor channels were applied, thus the calculated parameters are only related to stoichiometry and FRET efficiency but not the correct values (*11*, *12*).

Example scripts and example data that implement the described analysis pipelines are published openly in Zenodo (https://zenodo.org/records/14002224).

Supplementary Results

Spectroscopy

Our software offers high-level and low-level processing and analysis methods that can be flexibly combined to custom analysis pipelines and new analysis methodologies. The software can process and analyze ensemble fluorescence spectroscopy, SMS, and FIS data. For SMS photon traces can be filtered intuitively (*13*). High precision FRET analysis of photon histograms that takes data shot-noise explicitly into account (*14*) is enabled by scriptable photon distribution analysis (PDA) (*15*, *16*). Micro time information encoded in fluorescence data streams can be analyzed using maximum likelihood estimators for resolving model parameters such as anisotropies (*17*) and fluorescence lifetimes (*18*) at low photon counts for SMS and FIS analysis of bursts, and pixels. Model-free analysis through the phasor approach visualizes heterogeneity (*6*) for mapping metabolic states in cells by FIS(*6*, *19*). Fluorescence decay models consider experimental nuisances such as pile-up (*20*) and use CPU vector extensions for fast computation of complex models. FCS enables studying dynamic systems and informs on sample heterogeneity, diffusion coefficients, binding events, or intra-molecular dynamics (*21*). We compute fluorescence correlation spectroscopy curves for the registered photon stream using efficient algorithms (*22*). The implemented spectroscopic methods and algorithms can be applied to SMS and FIS data.

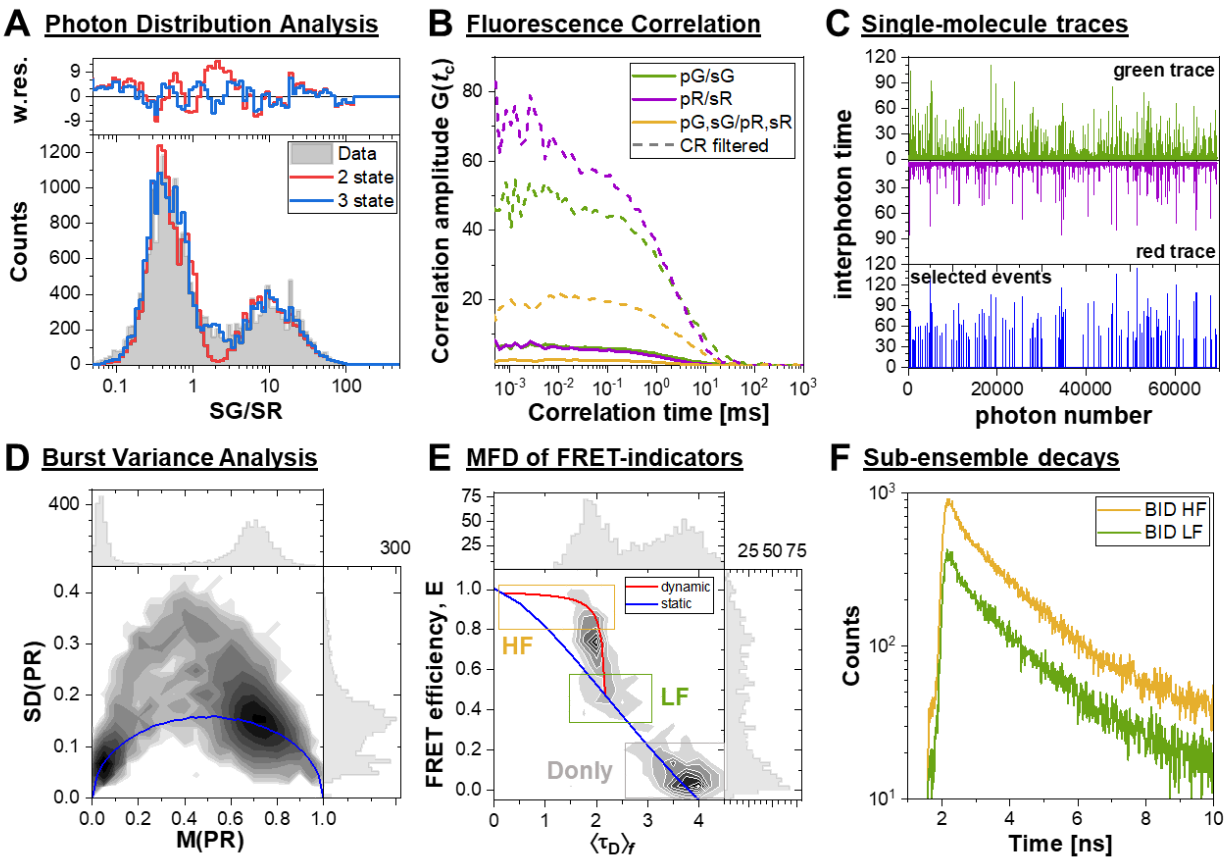

Supplementary Figure 1. Single molecule spectroscopy for dynamic structural biology on hGBP1. (A) For photon distribution analysis, the intensity traces were sliced into time windows (TW) of 1 ms, for TWs with at least 20 photons, the green-to-red intensity ratio Sg/Sr was calculated and Sg/Sr frequency histograms generated. The obtained PDA histogram (gray) was fitted either with a two state (red) or three state model (blue) including an additional donor-only population. Weighted residuals are shown on top. **(B)** Fluorescence correlation curves calculated either based on the whole intensity trace (solid lines) or when selecting only high-intensity regions as shown in (B) (dashed lines). For the count-rate filtered curves, only those regions of the intensity trace were selected where more than 60 photons per 10 ms time window have been detected. Green curves show the correlation of the two donor channels (pG/sG), magenta of the two acceptor channels (pR/sR) while in yellow the cross-correlation of the donor with the acceptor channel is shown (pG,sG/pR,sR). **(C)** Short section of a single-molecule time trace of freely diffusing hGBP1 molecules labeled with Alexa488-maleimide (donor) and Alexa647-maleimide (acceptor) at positions 344 and 525 . Top two panels show the photon detection event number vs the number of photons detected in the donor (green) or acceptor (magenta) channels per 1 µs time window. Bottom panel (blue) shows the selected photons, where a single-molecule burst was defined as the presence of more than 40 photons per 250 µs time window. **(D)** Burst variance analysis. The burstwise mean proximity ratio, $M(PR)$, and standard deviation (SD) of PR are shown in two-dimensional histograms (center) with one-dimensional projections of $M(PR)$ (top) and $SD(PR)$ (right). **(E)** The corrected FRET-efficiency, E, and fluorescence lifetime of the donor in the presence of A, $\langle \tau D \rangle f$, are shown in two-dimensional MFD-histograms (center) with one-dimensional projections of E (right) and $\langle \tau D \rangle f$ (top). The blue and the red line are so-called FRET-line, the theoretical function connecting static, ie, non-dynamic populations, and protein dynamics, respectively. Two double-labeled exchanging species can be identified: the low FRET species (green) and the high FRET species (yellow). **(F)** Donor fluorescence decay histograms computed for selected HF and LF bursts shown in (E).

We use a subset of the features implemented in our software in a single-molecule (sm) analysis pipeline to process human guanylate binding protein (hGBP1) smFRET data. In the confocal experiments, fluorescence of freely diffusing labeled hGBP1 in dilute solutions was registered (*1*). The analysis pipeline (*i*) reads smFRET data, (*ii*) selects single-molecule events, (*iii*) performs a burst analysis that computes intensity and lifetime-based FRET indicators, (*iv*) uses filters to correlate the photon traces, (*v*) generates single-molecule counting histograms, that are (*vi*) analyzed by photon distribution analysis (PDA), and (*vii*) selects molecular sub-ensembles,

A set of filters can be applied to the data before correlation. A photon stream can be filtered based on the macro (*23*) or the micro time information (*24–27*). We provide examples for the most common correlation approaches such as intensity filtered correlation, micro time gated correlation, and lifetime filtered correlations in the online documentation. By combining the base functionality with slicing of the photon stream into chunks with correlation methods automated robust FCS analysis can be used for live-cell measurements (*28*) and was as previously implemented for the β2-adrenergic receptor (*29*).

Using the fluorescence intensity of different detection channels fluorescence intensity counting histograms that can be used to determine FRET efficiencies (*15*) or fluorescence anisotropy (*30*) by PDA (**Supplementary Figure 1A**). The photon traces obtained in a single-molecule of FCS experiment can be correlated in a software correlator that can use filters (*31*), *e.g.*, based on the photon macro and micro time (**Supplementary Figure 1B**). In confocal smFRET experiments labeled molecules give rise to bursts in photon traces (**Supplementary Figure 1C**). The photon traces can be binned and count rate filters are used to select windows that exceed a user-defined threshold value in the average count rate to select single molecule bursts (*13*). The photons in a burst are integrated to give average count rates. Burst-variance analysis (BVA) (*2*) can be used to identify conformational dynamics within proteins (*32*) by computing the mean and the standard deviation of (*PR*) and comparing it to the shot-noise dynamics can be identified (*3*) **(Supplementary Figure 1D**). Here, we compute BVA histograms for the hGBP1 variant Q323C-Q525C. BVA highlights transitions from FRET to no FRET states (**Supplementary Figure 1D**). We use the count rates in the green and the red detection channels

that detect fluorescence light of donor and acceptor fluorophores in a FRET experiment to compute the FRET proximity ratio (*PR*) and the corrected FRET efficiency (*E*) for every burst (*33*). Next, we determine for every bust the fluorescence weighted average lifetimes and a corrected FRET efficiency to compute MFD histograms. The data was acquired on a MFD setup where the sample is excited by polarized light and the emitted light by the sample is split into parallel and perpendicular detection channels. Thus, we moreover analyze the fluorescence in the two channels to obtain anisotropies (*17*). In our analysis we analyze the micro times of photons in a burst using an MLE estimator to determine fluorescence averaged lifetimes (*18*). Thus, for each molecule we determine anisotropies, fluorescence lifetimes, and fluorescence intensities. Using these observables we compute multi-dimensional histograms (**Supplementary Figure 1E**). Using the fluorescence observables as features, single-molecules can be classified and grouped (**Supplementary Figure 1E**) to resolve species by fluorescence decays of sub-ensembles (**Supplementary Figure 1F**). For Q323C-Q525C we identify three populations: a high FRET population (HF), a low FRET population (LF), and molecules lacking an acceptor molecule (donor-only, DO). The FRET molecules are not described by the static FRET line (**Supplementary Figure 1E**, blue line) but is however described by a dynamic FRET line (**Supplementary Figure 1E**, red line) that describes the exchange between HF and LF states (*3*). The single molecule burst can be selected based and grouped into sub-ensembles, *e.g.*, for computing sub-ensemble fluorescence decay histograms (**Supplementary Figure 1F**).

Image fluorescence spectroscopy

We present an image spectroscopy (FIS) pipeline (**Supplementary Figure 2**) that processes Multiparameter fluorescence image spectroscopy (MFIS) PIE time series acquired on MEF cells transfected with murine guanylate binding proteins 3 (mGBP3) and mGBP7 N-terminally tagged with mCherry and eGFP, respectively. GBPs are primarily localized in the cytoplasm and accumulate in vesicle-like structures (VLS) (*5*, *34*). The pipeline: (*i*) groups photons into pixels of intensity images, (*ii*) performs a typical FLIM analysis for multiple detection channels and excitation sources, (*iii*) uses typical image preprocessing for segmentation of the image into pixel classes, (*iv*) uses the model free phasor approach to highlight sample heterogeneity, and (*v*) extracts fluorescence decays of pixel-classes for sub-ensemble analysis.

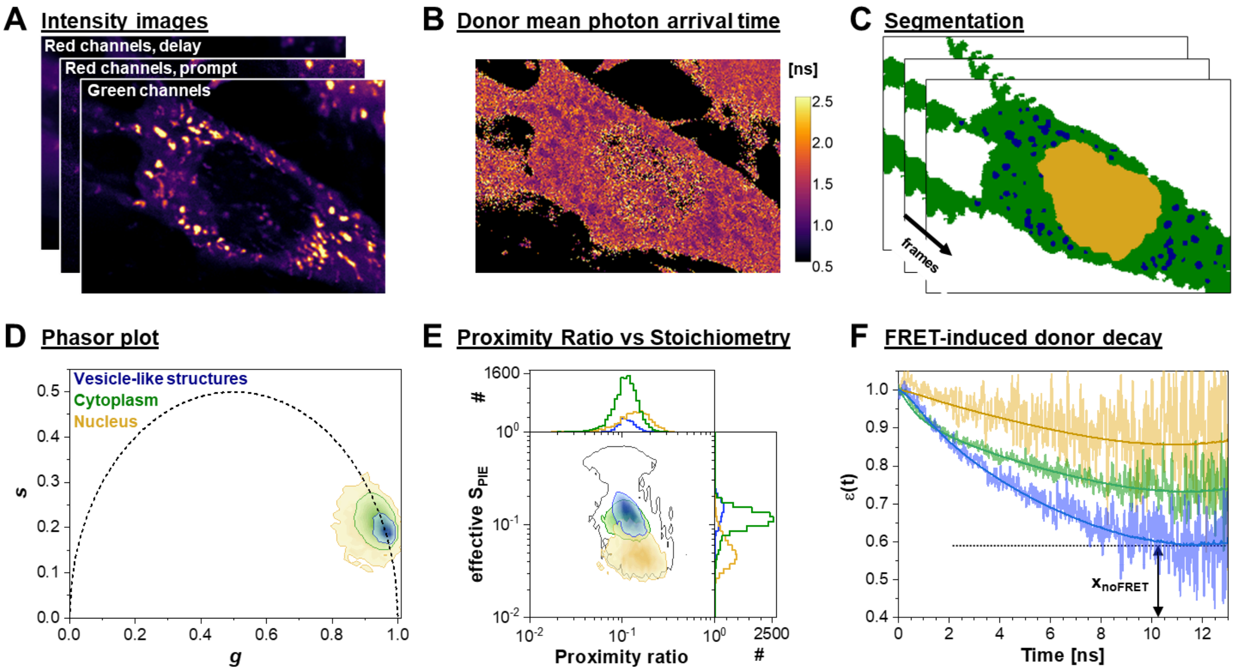

Supplementary Figure 2. PIE-MFIS FLIM dataset of an MEF cell transfected with mGBP-eGFP and mGBP-mCherry. (A) Intensity images of the donor (eGFP; green prompt) and acceptor (mCherry; red prompt / red delay) detection channels. The total intensity is calculated from the sum of the micro time events in the specified prompt and delay time windows. Shown is the sum over all acquired frames. **(B)** Donor mean-photon arrival time calculated from the micro time distribution for the stacked frames. Only pixels with more than three detected photons per frame are considered. **(C)** Frame-wise segmentation of intensity images into three pixel classes: cytoplasm (green), vesicle-like structures (VLS, blue) and nucleus (yellow). **(D)** Phasor analysis of the donor signal (prompt time window) for the specified cellular compartments. **(E)** Pixel-wise calculation of the apparent FRET efficiency and stoichiometry for each of the three pixel classes. Marginal projections of the parameter distributions are shown on the top and right, respectively. **(F)** FRET-induced donor decay was extracted based on the photon arrival time histograms of the pixel classes defined in (E) and a reference measurement of an eGFP-only transfected cell.

We compute intensity images for the signal detected by the green detector when the sample is excited by the green light source, G|G, the red detector when the sample is excited by the green light source, R|G, and the red detector for the sample excited by the red light source, R|R. The localization of GBPs in the cytoplasm and their accumulation in VLS is visible in intensity images (**Supplementary Figure 2A**). For the photons detected in the green channel and each pixel we determine an average fluorescence lifetime, τ_G, (*18*) (**Supplementary Figure 2B**) and the anisotropy, r_G (*17*). These features can be used for classifying pixels (*35*). We classify each pixel in each frame to either the cytoplasm (green), VLS (blue), or the nucleus (green) to study FRET location specific (**Supplementary Figure 2C**). The model free phasor approach

highlights sample heterogeneities (**Supplementary Figure 2D**). Such heterogeneities are expected as GBPs form higher-order oligomers in VLS and are monomeric and dimeric in the cytoplasm (*4*). For each pixel in a pixel class, we compute the proximity ratio and the effective stoichiometry. Proximity ratios and effective stoichiometries are computed using the uncorrected G|G, R|G, and R|R signal intensities. The effective stoichiometry, S^{PIE}, is a measure for the donor/acceptor ratio and the proximity ratio relates to the FRET efficiency (*11*). For the cytoplasm (green), VLS (blue), and the nucleus (green) we find distinct differences (**Supplementary Figure 2E**). Fluorescence decays computed for entire images or for groups of pixels can be computed for fluorescence decay analysis and visualized using the extracted FRET-induced donor decay, ε(t). Fluorescence decays of corresponding pixel classes can serve as input for later integrative modeling (*36*).

References

1. T.-O. Peulen, C. S. Hengstenberg, R. Biehl, M. Dimura, C. Lorenz, A. Valeri, J. Folz, C. A. Hanke, S. Ince, T. Vöpel, B. Farago, H. Gohlke, J. P. Klare, A. M. Stadler, C. A. M. Seidel, C. Herrmann, Integrative dynamic structural biology unveils conformers essential for the oligomerization of a large GTPase. *eLife* **12**, e79565 (2023).

2. J. P. Torella, S. J. Holden, Y. Santoso, J. Hohlbein, A. N. Kapanidis, Identifying molecular dynamics in single-molecule FRET experiments with burst variance analysis. *Biophys. J.* **100**, 1568–1577 (2011).

3. A. Barth, O. Opanasyuk, T.-O. Peulen, S. Felekyan, S. Kalinin, H. Sanabria, C. A. M. Seidel, Unraveling multi-state molecular dynamics in single-molecule FRET experiments. I. Theory of FRET-lines. *J. Chem. Phys.* **156**, 141501 (2022).

4. E. Kravets, D. Degrandi, Q. Ma, T.-O. Peulen, V. Klümpers, S. Felekyan, R. Kühnemuth, S. Weidtkamp-Peters, C. A. Seidel, K. Pfeffer, Guanylate binding proteins directly attack Toxoplasma gondii via supramolecular complexes. *Elife* **5** (2016).

5. N. Steffens, C. Beuter-Gunia, E. Kravets, A. Reich, L. Legewie, K. Pfeffer, D. Degrandi, Essential Role of mGBP7 for Survival of Toxoplasma gondii Infection. *MBio* **11** (2020).

6. M. A. Digman, V. R. Caiolfa, M. Zamai, E. Gratton, The phasor approach to fluorescence lifetime imaging analysis. *Biophys. J.* **94**, L14–6 (2008).

7. S. van der Walt, J. L. Schönberger, J. Nunez-Iglesias, F. Boulogne, J. D. Warner, N. Yager, E. Gouillart, T. Yu, scikit-image contributors, scikit-image: image processing in Python. *PeerJ* **2**, e453 (2014).

8. N. Otsu, A Threshold Selection Method from Gray-Level Histograms. *IEEE Trans. Syst. Man*

Cybern. **9**, 62–66 (1979).

9. C. H. Li, C. K. Lee, Minimum cross entropy thresholding. *Pattern Recognit.* **26**, 617–625 (1993).

10. C. H. Li, P. K. S. Tam, An iterative algorithm for minimum cross entropy thresholding. *Pattern Recognit. Lett.* **19**, 771–776 (1998).

11. B. Hellenkamp, S. Schmid, O. Doroshenko, O. Opanasyuk, R. Kühnemuth, S. Rezaei Adariani, B. Ambrose, M. Aznauryan, A. Barth, V. Birkedal, M. E. Bowen, H. Chen, T. Cordes, T. Eilert, C. Fijen, C. Gebhardt, M. Götz, G. Gouridis, E. Gratton, T. Ha, P. Hao, C. A. Hanke, A. Hartmann, J. Hendrix, L. L. Hildebrandt, V. Hirschfeld, J. Hohlbein, B. Hua, C. G. Hübner, E. Kallis, A. N. Kapanidis, J.-Y. Kim, G. Krainer, D. C. Lamb, N. K. Lee, E. A. Lemke, B. Levesque, M. Levitus, J. J. McCann, N. Naredi-Rainer, D. Nettels, T. Ngo, R. Qiu, N. C. Robb, C. Röcker, H. Sanabria, M. Schlierf, T. Schröder, B. Schuler, H. Seidel, L. Streit, J. Thurn, P. Tinnefeld, S. Tyagi, N. Vandenberk, A. M. Vera, K. R. Weninger, B. Wünsch, I. S. Yanez-Orozco, J. Michaelis, C. A. M. Seidel, T. D. Craggs, T. Hugel, Precision and accuracy of single-molecule FRET measurements—a multi-laboratory benchmark study. *Nat. Methods* **15**, 669–676 (2018).

12. E. Lerner, A. Barth, J. Hendrix, B. Ambrose, V. Birkedal, S. C. Blanchard, R. Börner, H. Sung Chung, T. Cordes, T. D. Craggs, A. A. Deniz, J. Diao, J. Fei, R. L. Gonzalez, I. V. Gopich, T. Ha, C. A. Hanke, G. Haran, N. S. Hatzakis, S. Hohng, S.-C. Hong, T. Hugel, A. Ingargiola, C. Joo, A. N. Kapanidis, H. D. Kim, T. Laurence, N. K. Lee, T.-H. Lee, E. A. Lemke, E. Margeat, J. Michaelis, X. Michalet, S. Myong, D. Nettels, T.-O. Peulen, E. Ploetz, Y. Razvag, N. C. Robb, B. Schuler, H. Soleimaninejad, C. Tang, R. Vafabakhsh, D. C. Lamb, C. A. Seidel, S. Weiss, FRET-based dynamic structural biology: Challenges, perspectives and an appeal for open-science practices. *Elife* **10** (2021).

13. J. R. Fries, L. Brand, C. Eggeling, M. Köllner, C. A. M. Seidel, Quantitative Identification of Different Single Molecules by Selective Time-Resolved Confocal Fluorescence Spectroscopy. *J. Phys. Chem. A* **102**, 6601–6613 (1998).

14. S. Kalinin, T. Peulen, S. Sindbert, P. J. Rothwell, S. Berger, T. Restle, R. S. Goody, H. Gohlke, C. A. M. Seidel, A toolkit and benchmark study for FRET-restrained high-precision structural modeling. *Nat. Methods* **9**, 1218–1225 (2012).

15. M. Antonik, S. Felekyan, A. Gaiduk, C. A. M. Seidel, Separating structural heterogeneities from stochastic variations in fluorescence resonance energy transfer distributions via photon distribution analysis. *J. Phys. Chem. B* **110**, 6970–6978 (2006).

16. S. Kalinin, A. Valeri, M. Antonik, S. Felekyan, C. A. M. Seidel, Detection of structural dynamics by FRET: a photon distribution and fluorescence lifetime analysis of systems with multiple states. *J. Phys. Chem. B* **114**, 7983–7995 (2010).

17. J. Schaffer, A. Volkmer, C. Eggeling, V. Subramaniam, G. Striker, C. A. M. Seidel, Identification of Single Molecules in Aqueous Solution by Time-Resolved Fluorescence Anisotropy. *J. Phys. Chem. A* **103**, 331–336 (1999).

18. M. Maus, M. Cotlet, J. Hofkens, T. Gensch, F. C. De Schryver, J. Schaffer, C. A. Seidel, An experimental comparison of the maximum likelihood estimation and nonlinear least-squares

fluorescence lifetime analysis of single molecules. *Anal. Chem.* **73**, 2078–2086 (2001).

19. K. Perinbam, J. V. Chacko, A. Kannan, M. A. Digman, A. Siryaporn, A Shift in Central Metabolism Accompanies Virulence Activation in Pseudomonas aeruginosa. *mBio* **11** (2020).

20. W. Becker, Ed., *Advanced Time-Correlated Single Photon Counting Applications* (Springer, Cham, 2015).

21. E. L. Elson, D. Magde, Fluorescence correlation spectroscopy. I. Conceptual basis and theory. *Biopolymers* **13**, 1–27 (1974).

22. M. Wahl, I. Gregor, M. Patting, J. Enderlein, Fast calculation of fluorescence correlation data with asynchronous time-correlated single-photon counting. *Opt. Express* **11**, 3583–3591 (2003).

23. T. A. Laurence, A. N. Kapanidis, X. Kong, D. S. Chemla, S. Weiss, Photon Arrival-Time Interval Distribution (PAID): A Novel Tool for Analyzing Molecular Interactions. *J. Phys. Chem. B* **108**, 3051–3067 (2004).

24. S. Felekyan, S. Kalinin, H. Sanabria, A. Valeri, C. A. M. Seidel, Filtered FCS: species auto- and cross-correlation functions highlight binding and dynamics in biomolecules. *Chemphyschem* **13**, 1036–1053 (2012).

25. S. Felekyan, H. Sanabria, S. Kalinin, R. Kühnemuth, C. A. M. Seidel, Analyzing Förster resonance energy transfer with fluctuation algorithms. *Methods Enzymol.* **519**, 39–85 (2013).

26. P. Kapusta, M. Wahl, A. Benda, M. Hof, J. Enderlein, Fluorescence lifetime correlation spectroscopy. *J. Fluoresc.* **17**, 43–48 (2007).

27. M. Böhmer, M. Wahl, H.-J. Rahn, R. Erdmann, J. Enderlein, Time-resolved fluorescence correlation spectroscopy. *Chem. Phys. Lett.* **353**, 439–445 (2002).

28. J. Ries, M. Bayer, G. Csúcs, R. Dirkx, M. Solimena, H. Ewers, P. Schwille, Automated suppression of sample-related artifacts in Fluorescence Correlation Spectroscopy. *Opt. Express* **18**, 11073 (2010).

29. A. Balakrishnan, K. Hemmen, S. Choudhury, J.-H. Krohn, K. Jansen, M. Friedrich, G. Beliu, M. Sauer, M. J. Lohse, K. G. Heinze, Unraveling the hidden temporal range of fast β-adrenergic receptor mobility by time-resolved fluorescence. *Commun Biol* **5**, 176 (2022).

30. S. Kalinin, S. Felekyan, M. Antonik, C. A. M. Seidel, Probability distribution analysis of single-molecule fluorescence anisotropy and resonance energy transfer. *J. Phys. Chem. B* **111**, 10253–10262 (2007).

31. O. Opanasyuk, A. Barth, T.-O. Peulen, S. Felekyan, S. Kalinin, H. Sanabria, C. A. M. Seidel, Unraveling multi-state molecular dynamics in single-molecule FRET experiments. II. Quantitative analysis of multi-state kinetic networks. *J. Chem. Phys.* **157**, 031501 (2022).

32. N. Kolimi, J. Ballard, T. Peulen, R. Goutam, F. X. Duffy 3rd, C. A. Ramírez-Sarmiento, J. Babul, E. Medina, H. Sanabria, DNA controls the dimerization of the human FoxP1 forkhead domain. *Cell Rep Phys Sci* **5** (2024).

33. E. Sisamakis, A. Valeri, S. Kalinin, P. J. Rothwell, C. A. M. Seidel, Accurate single-molecule FRET

studies using multiparameter fluorescence detection. *Methods Enzymol.* **475**, 455–514 (2010).

34. L. Legewie, J. Loschwitz, N. Steffens, M. Prescher, X. Wang, S. H. J. Smits, L. Schmitt, B. Strodel, D. Degrandi, K. Pfeffer, Biochemical and structural characterization of murine GBP7, a guanylate binding protein with an elongated C-terminal tail. *Biochem. J* **476**, 3161–3182 (2019).

35. M. Somssich, Q. Ma, S. Weidtkamp-Peters, Y. Stahl, S. Felekyan, A. Bleckmann, C. A. M. Seidel, R. Simon, Real-time dynamics of peptide ligand-dependent receptor complex formation in planta. *Sci. Signal.* **8**, ra76 (2015).

36. C. A. Hanke, J. D. Westbrook, B. M. Webb, T.-O. Peulen, C. L. Lawson, A. Sali, H. M. Berman, C. A. M. Seidel, B. Vallat, Making fluorescence-based integrative structures and associated kinetic information accessible. *Nat Methods*, doi: 10.1038/s41592-024-02428-x (2024).
